# Atlantic-wide connectivity of Ascension Island green turtles revealed by finer-scale mitochondrial DNA markers

**DOI:** 10.1007/s10592-025-01720-3

**Published:** 2025-08-22

**Authors:** Sophia A. Coveney, Eva Jiménez-Guri, Samantha Ball, Nathalie Mianseko, Annette C. Broderick, Brendan J. Godley, Joana M. Hancock, Welton Quirino Pereira, Aissa Regalla, Rita Gomes Rocha, Cheibani Senhoury, Benoit de Thoisy, Dominic Tilley, Sarah Maria Vargas, Sam B. Weber, Ana Rita Patrício

**Affiliations:** 1 Centre for Ecology and Conservation, University of Exeter, Penryn, Cornwall, UK; 2 Department of Biosciences, Swansea University, Swansea, Wales UK; 3 Ascension Island Government Conservation and Fisheries Directorate, South Atlantic Ocean, Georgetown, Ascension Island UK; 4 Pointe Noire, Association Renatura Congo, Ecocentre, Republic of Congo; 5 Sharjah Marine Science Research Centre, University of Khorfakkan, Khorfakkan, UAE; 6 Olive Ridley Turtle Project, 91 Padiham Road, Sabden, Clitheroe, Lancashire, UK; 7 cE3c - Center for Ecology, Evolution and Environmental Changes & CHANGE - Global Change and Sustainability Institute, Faculdade de Ciências da, Universidade de Lisboa, Lisbon, Portugal; 8 Departamento de Ciências Biológicas, Universidade Federal do Espírito Santo, Vitória, Espírito Santo Brazil; 9 Instituto da Biodiversidade e Áreas Protegidas, Dr. Alfredo Simão da Silva (IBAP), Bissau, Guinea-Bissau; 10 CIBIO/InBio - Centro de Investigaçãoem Biodiversidade e Recursos Genéticos, Universidade do Porto, Vairão, Portugal; 11 BIOPOLIS Program in Genomics, Biodiversity and Land Planning, CIBIO, Campus de Vairão, Vairão, 4485-661 Portugal; 12 Parc National du Banc d’Arguin, Chami, Mauritanie; 13 Kwata NGO, Cayenne, French Guiana

**Keywords:** mtDNA, *Chelonia mydas*, mtSTR, Population genetics, Mixed stock analysis, Sea turtles

## Abstract

Marine turtles undertake long migrations across different geographies and habitats, exposing them to a wide range of threats throughout their lifespan. Analysing population structure and connectivity is key to informing effective conservation management. We expand knowledge of Atlantic-wide connectivity of green turtles (*Chelonia mydas*) by characterising the genetic structure of the Ascension Island nesting population, one of the largest in the Atlantic Ocean, and carrying out Atlantic population structure and mixed stock analyses using high-resolution genetic markers. We amplified a ~ 738 bp fragment (extended D-loop) and a highly polymorphic mitochondrial short tandem repeat (mtSTR) fragment of the mitochondrial DNA control region, designating haplotypes based on (1) extended D-loop and (2) the extended D-loop and mtSTR combined. Overall, 11 extended D-loop and 33 combined haplotypes were found, the dominant haplotypes being CM-A8.1 and CM-A8.1/7-12-4-4. Population structure analysis found three main genetic groups: Northwest Atlantic, Northern South America, and South and East Atlantic. Mixed stock analyses indicate Ascension Island as a major source for juvenile foraging aggregations in the Southwest Atlantic (34–55%) and Central Africa (18–78%), with some contribution to West Africa (3–20%). Green turtles are vulnerable to fishery bycatch in the coastal waters of the South and East Atlantic. Our study underlines how improving sample sizes of Atlantic mtSTR haplotypes could further elucidate green turtle connectivity across threatened regions. We urge international collaboration to minimise mtSTR data gaps, in order to enhance connectivity assessments and improve conservation measures between countries that share populations.

**Supplementary Information:**

The online version contains supplementary material available at 10.1007/s10592-025-01720-3.

## Introduction

Migratory marine species are important within marine ecosystems as they can provide many ecosystem services, namely nutrient storage and transport, community shaping through organism dispersal, trophic-dynamic regulations of populations, and biodiversity promotion (Ferretti et al. 2010; Tavares et al. 2019). Additionally, many of the migratory marine species are of conservation or commercial value, including tuna, seabirds, sharks, marine mammals, and marine turtles (Lascelles et al. 2014). These taxa often undertake long migrations across different habitats and distant geographical areas to reproduce, feed or develop, and during these extensive movements they can be vulnerable to a diverse range of threats. Thus, understanding dispersal, migratory movements and how populations are linked is crucial to perceiving threats, understanding their consequences, and informing effective management strategies (Martin et al. 2007; Wallace et al. 2011; Dunn et al. 2019).

Marine turtles are highly migratory, long-lived organisms, able to connect ocean basins throughout their life cycle (Boyle et al. 2009). After hatching, green turtles (*Chelonia mydas*) disperse in the open ocean and undergo an epipelagic omnivorous lifestyle for around 3–5 years (Reich et al. 2007). This phase is often known as ‘the lost years’ due to poor knowledge of their whereabouts (Carr 1980; Reich et al. 2007), although recent work has provided new insights (e.g., Mansfield et al. 2021). Following this period, juveniles generally recruit to coastal habitats and transition to benthic foraging (Bjorndal 1997). Juveniles can spend several years in the same feeding grounds, until reaching a certain size or a maturity point that triggers migration to alternative neritic foraging areas (Lenz et al. 2017) from where natal homing is undertaken. Adults of both sexes begin to periodically migrate between neritic foraging grounds and natal nesting sites (Bowen and Karl 2007). This philopatric behaviour can result in connections spanning large distances (Carr 1964; Patrício et al. 2017a). Techniques such as satellite telemetry, ocean current modelling and stable isotope analysis have all contributed to the knowledge of migratory connectivity in marine turtles (e.g., Godley et al. 2010; Seminoff et al. 2012; Scott et al. 2014; Putman and Mansfield 2015; Bradshaw et al. 2017; Ng et al. 2018; Ferreira et al. 2020; Kot et al. 2022), but molecular genetics has played a particularly key role, especially when assessing whole life cycle connectivity (e.g., Naro-Maciel et al. 2017; Jensen et al. 2020; Phillips et al. 2022). The combination of these techniques led to the creation of regional management units (RMUs; Wallace et al. 2010; recently updated in Wallace et al. 2023) that group together connected rookeries and foraging areas for management purposes.

Mitochondrial DNA (mtDNA) is a maternally inherited genetic marker that carries information on population structure (Harrison 1989). Haplotypes identified from the D-loop within the mtDNA control region have been used extensively in studies assessing marine turtle population structure and connectivity (e.g., Formia et al. 2007; Proietti et al. 2012; Shamblin et al. 2015b, 2018b; Patrício et al. 2017a, b; Jensen et al. 2020). These studies have demonstrated limited maternal gene flow among rookeries, with high levels of genetic structuring established along several female lineages, supporting the natal homing hypothesis wherein female turtles return to their natal beach to nest (Meylan et al. 1990). Juvenile foraging aggregations, on the other hand, are typically made up of a mixed stock of individuals from multiple nesting populations. Because rookeries are genetically structured, mixed stock analysis (MSA; Millar 1987) can be used to estimate how much a particular rookery contributes to a foraging aggregation and thus reveal how different rookeries and foraging aggregations are connected across the global ocean.

The population genetic structure of green turtles has been extensively studied using mtDNA over the past few decades across the Indian, Pacific and Atlantic oceans (Bowen et al. 1992; Encalada et al. 1996; Dethmers et al. 2006; Bourjea et al. 2007; Jensen et al. 2019). As per the recent update to green turtle RMUs, Atlantic green turtles are divided into two RMUs: the North Atlantic and South Atlantic (Wallace et al. 2023). Over the last decade, population studies have identified three main genetic groups for nesting populations (Northwest Atlantic, Northern South America, and South Atlantic & West Africa; Patrício et al. 2017a), and three main genetic groups for foraging aggregations (Northwest Atlantic, Central Atlantic, and South Atlantic & West Africa; Patrício et al. 2017b), with each group broadly characterised by a common genetic haplotype. Genetic structure in Atlantic green turtles has primarily been inferred for mtDNA haplotypes based on the traditionally used marker, a ~ 486 base pair (bp) sequence within the mtDNA control region (Encalada et al. 1996; Formia et al. 2006). However, recent studies have developed a database of haplotypes based on polymorphisms within an extended ~ 817 bp fragment of this region (hereby referred to as ‘extended D-loop’) that contains the shorter fragment (Shamblin et al. 2012), and on another more variable region of mtDNA that has short tandem repeats of ‘AT’ nucleotides (hereby referred to as the mtSTR; Tikochinski et al. 2012). Both the extended D-loop and mtSTR fragments provide higher resolution markers, allowing more detailed insight into population structure (Shamblin et al. 2015b, 2018a; Karaman et al. 2022). MtSTR haplotyping has been widely used in Mediterranean green turtles to unravel previously unidentified genetic differentiation at significant geographic scales, considering the nesting distribution in the Mediterranean (Tikochinski et al. 2018; Karaman et al. 2022). However, corresponding data in the wider Atlantic is limited and mainly restricted to specific regions, namely rookeries in the Northwest Atlantic, the Caribbean and South Atlantic islands off Brazil (Shamblin et al. 2015b, 2020; Barbanti et al. 2019). To achieve a comprehensive assessment of green turtle connectivity at meaningful scales, more data are needed across the Atlantic.

Isolated in the South Atlantic, approximately 8 degrees south of the equator and midway between the continents of Africa and South America, Ascension Island hosts one of the most significant green turtle rookeries globally, with a population estimate of 14,840 nesting females overall and 28,000 nests per annum (Weber et al. 2014). Historically, the island was subjected to mass harvesting of nesting green turtles for consumption, and as a result, the population was depleted (Broderick et al. 2006). However, thanks to conservation efforts in Brazil (Marcovaldi et al. 2000) and the end of harvest in Ascension, it is now in recovery (Weber et al. 2014). Green turtles are listed as Globally Endangered according to the Red List of the International Union for Conservation of Nature (IUCN; Seminoff 2023), and whilst the South Atlantic population is classed as Least Concern, this is considered to be conservation-dependent (Broderick and Patrício 2019). Further, negative population trajectories can take many years to become apparent due to their long-lived nature and long generational times. Threats to green turtles persist for the South Atlantic subpopulation; for example, an increased number of strandings have been reported for green turtles on the Brazilian coast, with notable threats including interactions with artisanal fisheries and ingestion of solid waste (Nunes et al. 2023). On the other side of the Atlantic, interactions with artisanal fisheries remain a present threat (dos Santos et al. 2024; Cardona et al. 2025; Mestre et al. 2025).

Previous studies have found that the CM-A8 haplotype, the haplotype most prevalent throughout the South Atlantic, was dominant among Ascension Island nesting turtles (Bjorndal et al. 2006; Formia et al. 2006). Connectivity between Ascension Island and foraging grounds on the western Atlantic continental margins (within the aforementioned South Atlantic & West Africa genetic grouping) is well established. Tagging and satellite telemetry collected over several decades suggest that adult foraging areas are located exclusively along the Brazilian continental shelf (Luschi et al. 1998; Hays et al. 2002), while mixed stock analysis suggests that juvenile foraging areas extend further south into northern Argentina and Uruguay (Caraccio 2008; Prosdocimi et al. 2012) and as far north as the Caribbean (e.g., Luke et al. 2004). Links have also been proposed between Ascension Island and juvenile foraging grounds along the east coast of Africa, but low marker resolution and poor geographic coverage of sampling have, as yet, limited robust conclusions (Bolker et al. 2007; Patrício et al. 2017a). Obtaining mtSTR haplotypes for Ascension Island, and other South Atlantic populations, could help distinguish differences within the widely dominant CM-A8/CM-A8.1 haplotype.

Here, we reassess the structure of Atlantic green turtle rookeries, using the largest and most recent dataset of haplotypes based on the extended D-loop fragment (*n* = 21 rookeries). Using mixed stock analysis, we use the extended D-loop and mtSTR sequences to assess the contribution of Ascension Island to different South Atlantic foraging aggregations at a more robust level. We incorporate novel haplotype data from Ascension Island and other rookeries/foraging aggregations of green turtles from the South Atlantic RMU (Guinea-Bissau, Congo and Brazil; Wallace et al. 2010; Wallace et al. 2023). This study will provide an extensive and contemporary analysis of Atlantic-wide genetic structure and connectivity in order to better understand how green turtles are connected across countries bordering the Atlantic Ocean and inform the optimisation of conservation strategies.

## Materials and methods

### Study site and sampling

**Fig. 1:**
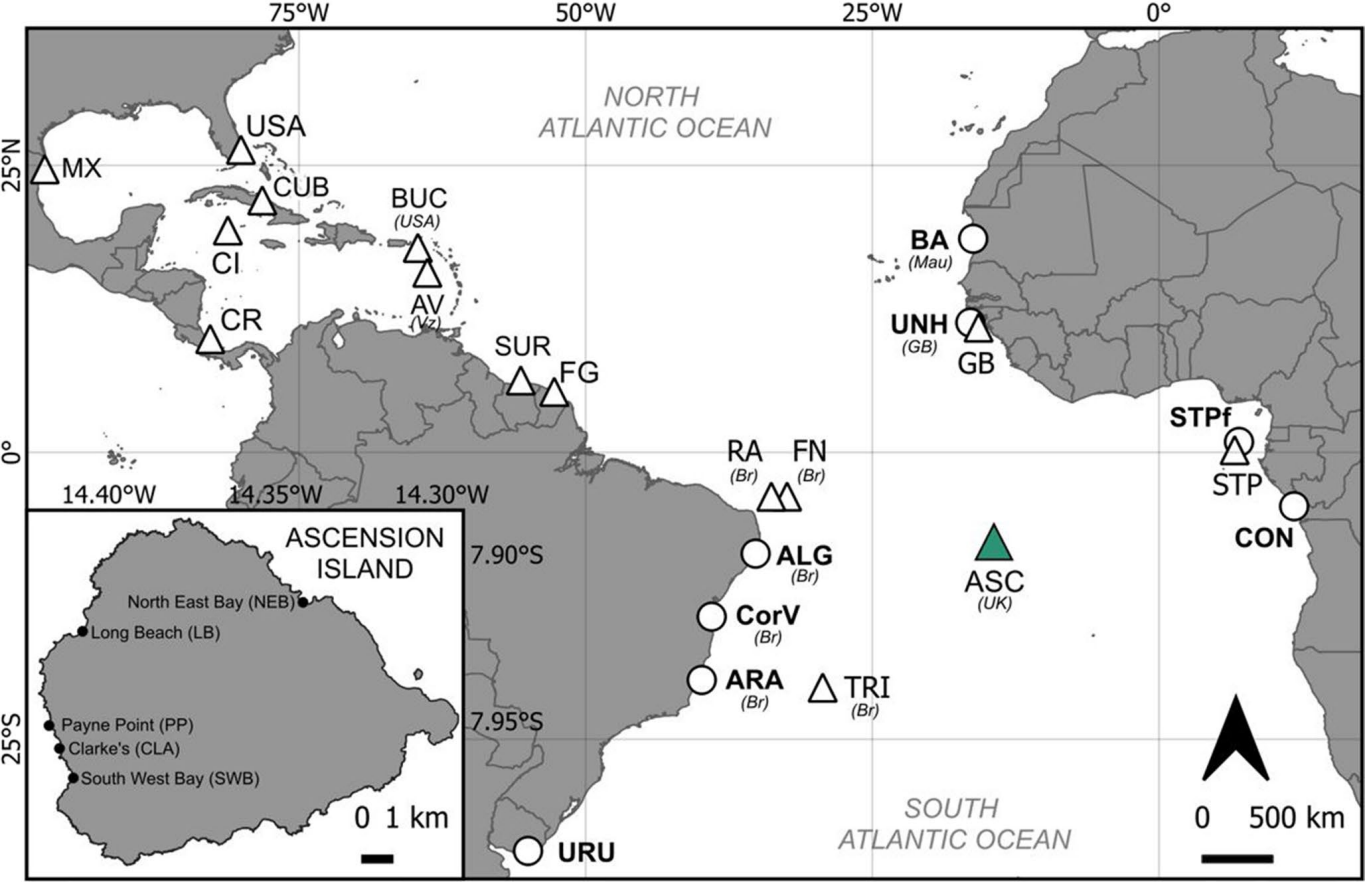
Location of Atlantic rookeries (triangles) and foraging grounds (circles) included in this study. The study site is in green. See Table 1 for site abbreviations. Br = Brazil, GB = Guinea-Bissau, Mau = Mauritania, UK = United Kingdom, USA = United States of America. Inset: Map of the study site, Ascension Island, UK. Beaches where samples were collected are labelled

Ascension Island is a United Kingdom overseas territory located in the centre of the South Atlantic Ocean (7.94° S, 14.36° W; Fig. 1). Green turtle biopsy samples (*n* = 303) were collected opportunistically during night surveys of nesting females in 2015 and 2016, across the months of January to May (peak nesting is in March; Godley et al. 2001). Samples were taken from the three main nesting beaches on the island, Long Beach (LB), North East Bay (NEB) and South West Bay (SWB), which together host around 75% of nesting (Weber et al. 2014), and two further beaches, Clarke’s and Payne Point, which host minimal nesting (Fig. 1, see Table S1 for the number of samples collected from each beach in each year). Turtles were sampled after oviposition to minimise disturbance of nesting behaviour. Soft tissue biopsies of ~ 5 mm diameter were taken from adult females from the epidermis of the neck area, or in some rare cases from the front flipper, and stored in 96% ethanol at ambient room temperature.

Tagging of individuals was not carried out; however, due to the high volume of nesting females and relatively low sampling effort, the probability of repeat sampling of the same individuals within a year was deemed negligible. Additionally, Ascension Island green turtles have an average remigration interval of four years (Mortimer and Carr 1987) and so sampling over two consecutive years most likely prevented repeat sampling across seasons.

### Sequencing and haplotype assignment

DNA was extracted from biopsy samples using the QIAGEN DNeasy Blood & Tissue Kit, according to the manufacturer’s instructions. The primers LCM15382 (5’-GCT TAA CCC TAA AGC ATT GG-3’) and H950 (5’-TCT CGG ATT TAG GGG TTT-3’; Abreu-Grobois et al. 2006) were used to amplify a ~ 860 bp fragment of the mtDNA control region (extended D-loop) using polymerase chain reaction (PCR). This fragment contained the ~ 486 bp short region which has typically been analysed in genetic studies of green turtles (Formia et al. 2006, 2007). The primers CM-D-1 F (5′-AGCCCATTT ACTTCT CGCCAAACCCC‐3′) and CM‐D‐5 R (5′‐GCTCCTTTTATCTGATGGG ACTGTT‐3′; Tikochinski et al. 2012) were used to amplify a ~ 200 bp short tandem repeat region at the end of the mtDNA control region (mtSTR).

PCRs were conducted in a total volume of 15 µl containing: 0.75 µl of each forward and reverse primer at 10 µM; 7.5 µl of QIAGEN’s Taq PCR Master Mix (contains Taq DNA Polymerase, 2 x QIAGEN PCR Buffer, 3 mM MgCl_2_, and 400 µM of each dNTP); 3 µl of ddH_2_O; and 3 µl of DNA at 10 µM. Cycling conditions for the mtDNA extended D-loop sequence were as follows: 94 °C for 5 min; 30 cycles of 94 °C for 1 min, 52 °C for 1 min and 72 °C for 1 min; then 72 °C for 10 min. For the mtSTR, conditions were: 94 °C for 2 min; 30 cycles of 94 °C for 30 s; 56 °C for 30 s, and 72 °C for 1 min; followed by 72 °C for 7 min. For certain samples that did not amplify effectively at 30 cycles, the number of cycles was increased to 35. The Cytiva ExoProStar one-step Exonuclease I and Alkaline Phosphatase solution was used to purify the PCR products required for sequencing, removing any unincorporated primers and dNTPs. Incubation for 15 min at 37 °C followed by 15 min at 80 °C was carried out to enable purification before inactivating the enzymes. Forward and reverse DNA strands were sequenced for the extended D-loop sequence, and for the mtSTR marker, only forward DNA strands were sequenced initially. When mtSTR regions retrieved an unclear sequencing result, we repeated the PCR, sequencing both the forward and reverse strands. Failed extended D-loop sequences were also repeated with both primers. Sequencing was carried out at Macrogen (Netherlands).

Sequence assemblage and manual alignment were carried out using BioEdit 7.2.5 (Hall 1999). The extended D-loop sequences were truncated to ~ 738 bp. The basic local alignment search tool (BLAST) from the National Centre for Biotechnology Information (www.ncbi.nlm.nih.gov) was used to identify haplotypes, following Archie Carr Center for Sea Turtle Research (ACCSTR; https://accstr.ufl.edu/resources/mtdna-sequences/) nomenclature.

MtSTR haplotypes were designated according to the number of ‘AT’ pairs present in 4 loci following Tikochinski et al. (2012), e.g., 7-12-4-4. In cases of heteroplasmy, chromatograms were used to identify the haplotype from the dominant peaks (Tikochinski et al. 2012) and in cases where this was unclear, the mtSTR sequence was removed from the study.

### Genetic composition

We truncated the control region fragment to the ~ 486 bp segment in order to compare the genetic composition of Ascension Island (*n* = 289) in this study to previous data from this island (Formia et al. 2007; *n* = 245), with pairwise comparisons based on frequency-based genetic distances (*F*_*ST*_) conducted in Arlequin 3.5.1.3 (Excoffier and Lischer 2010). Within the contemporary data, we also conducted pairwise comparisons (*F*_*ST*_) using the extended D-loop, and extended D-loop and mtSTR combined to examine whether there was significant genetic differentiation between the three main Ascension Island nesting beaches, i.e., South West Bay, North East Bay, and Long Beach. Clarke’s and Payne Point were excluded from this analysis due to the small sample size and relatively low nesting frequency (Weber et al. 2014).

**Table 1:** Atlantic nesting populations and foraging grounds used for the population structure analyses and mixed stock analyses in this study. Mixed stock analysis (MSA) rookery grouping is included for populations used in the mixed stock analyses. Regional management unit (RMU) refers to those designated by ( Wallace et al. 2023 )

Site name	Abbrev.	RMU	MSA group	Haplotype data	References (Haplotype data)
**Nesting populations**					
Northern US Limit (South Carolina, North Carolina, Delaware)	NUSA	North Atlantic	USA	Extended D-loop	(Shamblin et al. 2018a)
Central eastern Florida	CEFL	North Atlantic	USA	Extended D-loop + mtSTR	(Shamblin et al. 2020)
Southeastern Florida	SEFL	North Atlantic	USA	Extended D-loop + mtSTR	(Shamblin et al. 2020)
Key West, Florida	MKFL	North Atlantic	USA	Extended D-loop + mtSTR	(Shamblin et al. 2020)
Dry Tortugas, Florida	DTFL	North Atlantic	USA	Extended D-loop + mtSTR	(Shamblin et al. 2020)
Quintana Roo, Mexico	QRMX	North Atlantic	MX	Extended D-loop	(Pérez-Ríos 2008; Shamblin et al. 2018b)
Tamaulipas and Veracruz, Mexico	WBCMX	North Atlantic	MX	Extended D-loop	(Millán-Aguilar 2009; Shamblin et al. 2018b)
Campeche and Yucatan, Mexico	EBCMX	North Atlantic	MX	Extended D-loop	(Millán-Aguilar 2009; Shamblin et al. 2018b)
Cayo Arcas, Campeche, Mexico	CAMX	North Atlantic	MX	Extended D-loop	(Millán-Aguilar 2009; Shamblin et al. 2018b)
Scorpion Reef, Yucatan, Mexico	SRMX	North Atlantic	MX	Extended D-loop	(Millán-Aguilar 2009; Shamblin et al. 2018b)
Guanahacabibes Peninsula and San Felipe, Cuba	GUCB	North Atlantic	CUB	Extended D-loop	(Azanza-Ricardo et al. 2023)
Isla de la Juventud	IJCB	North Atlantic	CUB	Extended D-loop	(Azanza-Ricardo et al. 2023)
Cayo Largo, Cuba	CLCB	North Atlantic	CUB	Extended D-loop	(Azanza-Ricardo et al. 2023)
Tortuguero, Costa Rica	CR	North Atlantic	CR	Extended D-loop + mtSTR	(Shamblin et al. 2023a)
Grand Cayman (wild population), Cayman Islands	CI	North Atlantic	CI	Extended D-loop + mtSTR	(Barbanti et al. 2019)
Buck Island	BUC	North Atlantic	BUC	Extended D-loop	(Shamblin et al. 2012)
Aves Island, Venezuela	AV	North Atlantic	AV	Extended D-loop	(Shamblin et al. 2012)
Matapica and Galibi, Suriname	SUR	North Atlantic	SUR	Extended D-loop	(Shamblin et al. 2012)
Cayenne, French Guiana	FG	North Atlantic	FG	Extended D-loop + mtSTR	(Jordão et al. 2017; Patrício et al. 2024)
Atol das Rocas, Brazil	RA	South Atlantic	RA	Extended D-loop + mtSTR	(Shamblin et al. 2015b)
Fernando de Noronha, Brazil	FN	South Atlantic	FN	Extended D-loop + mtSTR	(Shamblin et al. 2015b)
Trindade Island, Brazil	TRI	South Atlantic	TRI	Extended D-loop + mtSTR	(Shamblin et al. 2015b)
Ascension Island, UK	ASC	South Atlantic	ASC	Extended D-loop + mtSTR	This study
Poilão, Guinea-Bissau	GB	South Atlantic	GB	Extended D-loop + mtSTR	(Patrício et al. 2024)
São Tomé and Príncipe	STP	South Atlantic	STP	Extended D-loop + mtSTR	(Hancock et al. 2019)
**Foraging grounds**					
Alagoas, Brazil	ALG	N/A	N/A	Extended D-loop	(Almeida et al. 2021)
Coroa Vermelha, Brazil	CorV	N/A	N/A	Extended D-loop + mtSTR	Laboratório de Genética e Evolução Molecular, UFES, unpublished data
Aracruz, Brazil	ARA	N/A	N/A	Extended D-loop + mtSTR	Laboratório de Genética e Evolução Molecular, UFES, unpublished data
Uruguay	URU	N/A	N/A	Extended D-loop	(Prosdocimi et al. 2024)
Congo	CON	N/A	N/A	Extended D-loop + mtSTR	Congo Renatura, unpublished data
São Tomé and Príncipe	STP	N/A	N/A	Extended D-loop + mtSTR	(Hancock et al. 2019; Patrício et al. 2024)
Unhocomo, Guinea-Bissau	UNH	N/A	N/A	Extended D-loop + mtSTR	This study
Parc National du Banc D’Arguin, Mauritania	BA	N/A	N/A	Extended D-loop + mtSTR	(Patrício et al. 2024)

### Population structure

We estimated the haplotype diversity (*h*) of green turtle Atlantic rookeries for the extended D-loop and for the extended D-loop combined with mtSTR haplotypes (*h*), in Arlequin 3.5.1.3 (Excoffier and Lischer 2010); see Table 1 for a list of rookeries used; see Table S2 for extended table including groupings used for MSAs and nester abundance data; see Table S3 and S4 for haplotype data). For the extended D-loop haplotypes, we estimated pairwise comparisons based on sequence divergence among haplotypes (*Ф*_*ST*_) and on haplotype frequencies (*F*_*ST*_) in Arlequin 3.5.1.3. For the extended D-loop and mtSTR combined, due to the nature of the mtSTR, only frequency-based statistics were considered (Tikochinski et al. 2018; Shamblin et al. 2023a). Because the South Atlantic region is dominated by the CM-A8 haplotype, additional *F*_*ST*_ comparisons were conducted using only CM-A8 combined with mtSTRs (CM-A8 + mtSTRs) to assess potential variation within mtSTRs linked to the dominant D-loop haplotype and to reduce noise from less common haplotypes. To calculate an optimum threshold for *P*-value significance, a false discovery rate (FDR) correction (Narum 2006) was applied, considering the total number of comparisons carried out in the analysis under an expected original threshold of *P* ≤ 0.05. The R package ‘gplots’ was used to create heatmaps with dendrograms based on *F*_*ST*_/*Ф*_*ST*_ values (Warnes et al. 2024) in RStudio using R version R-4.3.1 (RStudio Team 2020, R Core Team 2023). The software GenAlEx 6.51b2 (Peakall and Smouse 2006, 2012) was used to perform a principal coordinate analysis (PCoA), using the genetic distances to visualise how the rookeries were grouped genetically. Separate PCoAs were carried out for the extended D-loop and extended D-loop and mtSTR combined haplotype datasets. Using the results as a priori grouping, the significance was tested by carrying out an analysis of molecular variance (AMOVA) in Arlequin 3.5.1.3 (Excoffier and Lischer 2010).

### Mixed stock analyses

We used the mixstock package in R (Bolker et al. 2007) to conduct ‘many-to-many’ mixed stock analyses (MSAs; Okuyama and Bolker 2005; Bolker et al. 2007; Stahelin et al. 2022) to estimate the relative contribution from Ascension Island to eight juvenile green turtle foraging aggregations (see Table 1 for site names and locations) within the South Atlantic RMU (Wallace et al. 2010, 2023). Separate MSAs were conducted using the extended D-loop and the extended D-loop and mtSTR combined. Analyses were conducted with and without priors based on nester abundance. Nester abundance (Table S2), defined as the number of nesting females (Seminoff et al. 2015), was used to establish weighted priors. All analyses were run with 50,000 iterations. A Gelman-Rubin convergence diagnostic was applied (Gelman and Rubin 1992), and results indicated chain convergence if the shrink factor was < 1.2. As we aimed to specifically assess the contributions from Ascension Island, contributions from other rookeries and source-centric results are not presented or interpreted in this paper.

For the MSAs, individual rookeries within Florida, Cuba and Mexico (all outside of the South Atlantic RMU) were combined by country (Table 1), reflecting national boundaries and, hence, management. We know from previous studies that these populations have negligible importance to our research question (Naro-Maciel et al. 2007; Patrício et al. 2017a). We decided not to dismiss them from the mixed stock analysis due to the overlap of a few D-loop haplotypes between these rookeries and the foraging grounds assessed. The dataset for MSAs was composed of 15 rookery groups and 8 foraging aggregations for the extended D-loop and 10 rookery groups and 6 foraging aggregations for the extended D-loop and mtSTR combined (Table 1). The latter had fewer rookeries and foraging aggregations because mtSTR data is still missing from several sites. For foraging aggregation datasets, only turtles that fell within the size range of immature individuals were included. This avoids biased results in MSAs by including adult individuals that may have different dispersal patterns and be more prone to forage closer to home (Hays and Scott 2013). In foraging datasets not restricted to immature individuals upon data collection (Banc D’Arguin, Mauritania; Unhocomo, Guinea-Bissau; Alagoas, Brazil), adult life stage was designated based on curved carapace length (CCL) of nesting green turtles, using the average minimum adult size as a threshold. Average minimum adult size was calculated as the mean nesting female size minus two standard deviations (Stewart et al. 2007; Phillips et al. 2021). Satellite tracking and flipper tagging data have shown that breeding females from Poilão Island use foraging areas in Mauritania/Guinea-Bissau (Catry et al. 2023), whereas adult females from Ascension Island forage in Brazil (Luschi et al. 1998). Hence, adult size class for Banc d’Arguin and Unhocomo was calculated using CCL data from Poilão (*n* = 409, 2018–2021; Catry et al. 2023), and for Brazilian foraging grounds, data from Ascension Island were used (*n* = 788, 2012–2022; unpublished data from Ascension Island Government Conservation and Fisheries Directorate). Since we only had extended D-loop and mtSTR haplotypes from a single French Guiana beach, these were extrapolated to the entire country. Due to missing extended D-loop sequence information for some haplotypes within the Mexico nesting populations, assumptions of the most common associated extended sequence were made from shorter D-loop haplotype data. These assumptions enabled the inclusion of this population in the MSA and are not expected to significantly impact results, as the implicated haplotypes are absent from Ascension Island and from all South Atlantic foraging areas assessed except one (Unhocomo, Guinea-Bissau: CM-A26.1, 1.16%).

## Results

### Genetic composition

DNA extraction or amplification was successful for 289 nesting female tissue samples (95% of the samples), which were sent for sequencing. We found 10 different shorter (~ 486 bp) D-loop haplotypes, which separated into 11 extended (~ 738 bp) D-loop haplotypes. The CM-A8 shorter haplotype was dominant at Ascension Island (74.4% of the total sample set), but using the longer sequences we identified two variants: CM-A8.1 and CM-A8.3 (98.6% and 1.4% of total CM-A8 samples, respectively, Table 2). These higher-resolution characterisations also resulted in two previously unnamed haplotypes, CM-A45.1 (GenBank accession number: PP429908) and CM-A39.1 (PQ604655). We identified the haplotype CM-A42.1 for the first time in Ascension Island in four turtles (1.4%). CM-A69 (CM-A69.1, previously an orphan haplotype identified in São Francisco de Itabapoana, Brazil; Jordão et al. 2017) was also found (0.4%).

For the mtSTR, 19 different haplotypes were identified, of which 16 were present among individuals with CM-A8 haplotypes. The highest frequency mtSTR haplotype was 7-12-4-4 (55.4% of 202 successfully identified mtSTR). When considering extended D-loop sequences combined with the mtSTR, 33 haplotypes were identified, of which over half were CM-A8 variants (*n* = 18). For all extended D-loop haplotypes except CM-A8.3, CM-A39.1 and CM-A45.1, combinations with 7-12-4-4 were the most frequent. Heteroplasmy, wherein two mtDNA haplotypes are present within one individual, was present to some degree in all our mtSTR samples, and for 54 sequences, the dominant haplotype was deemed unclear (Table S5). These samples were excluded from analyses using mtSTR haplotypes. The genetic variability of the Ascension Island rookery was intermediate in comparison to the other Atlantic rookeries (Table 3).

**Table 2:** Haplotype frequencies derived from 289 DNA samples from Ascension Island green turtles (*Chelonia mydas).* Haplotypes based on both a ~ 486 bp and ~ 738 bp mitochondrial control region sequences (short and extended D-loop, respectively) and on ~ 200 bp short tandem repeats in the mtDNA control region (mtSTR) are shown. ‘Unidentified’ represents samples where D-loop haplotypes were identified, but the sequencing of the mtSTR region was indiscernible and did not enable haplotype identification due to either problematic sequencing or heteroplasmy. NA represents samples where D-loop haplotypes were not identified due to problematic sequencing

	Haplotype			Frequency	Percentage
486 bp	738 bp	Accession number	mtSTR		frequency
**CM-A6**	**CM-A6.1**	JQ366073	7-12-4-4	5	1.7
			Unidentified	2	0.7
**CM-A8**	**CM-A8.1**	JF308472	6-12-4-4	5	1.7
			6-13-4-4	9	3.1
			6-14-4-4	3	1.0
			6-17-4-4	3	1.0
			7-11-4-4	13	4.5
			7-12-4-4	85	29.4
			7-13-4-4	14	4.8
			7-14-4-4	1	0.3
			7-15-4-4	2	0.7
			7-16-4-4	6	2.1
			8-10-4-4	1	0.3
			8-11-4-4	7	2.4
			8-12-4-4	5	1.7
			8-13-4-4	1	0.3
			8-14-4-4	5	1.7
			8-15-4-4	1	0.3
			Unidentified	51	17.6
	**CM-A8.3**	JF308474	7-11-4-4	2	0.7
			7-12-4-4	1	0.3
**CM-A9**	**CM-A9.1**	JF308475	7-11-4-4	3	1.0
			7-12-4-4	5	1.7
			Unidentified	2	0.7
**CM-A10**	**CM-A10.1**	JF308476	7-12-4-4	2	0.7
			7-17-4-4	1	0.3
			8-12-4-4	1	0.3
			Unidentified	5	1.7
**CM-A24**	**CM-A24.1**	JF308479	7-12-4-4	6	2.1
**CM-A32**	**CM-A32.1**	JF308480	7-12-4-4	1	0.3
			Unidentified	1	0.3
**CM-A39**	**CM-A39.1**	PQ604655	7-12-4-4	2	0.7
			8-12-4-4	3	1.0
			Unidentified	1	0.3
**CM-A42**	**CM-A42.1**	JF308481	6-17-4-4	1	0.3
			7-12-4-4	2	0.7
			Unidentified	1	0.3
**CM-A45**	**CM-A45.1**	PP429908	5-13-4-4	1	0.3
			5-14-4-4	1	0.3
			Unidentified	5	1.7
**CM-A69**	**CM-A69.1**	KC792574	7-12-4-4	1	0.3
**NA**	**NA**		6-12-4-4	1	0.3
			7-12-4-4	2	0.7
			Unidentified	19	6.6
**Total**				289	100.0

**Fig. 2:**
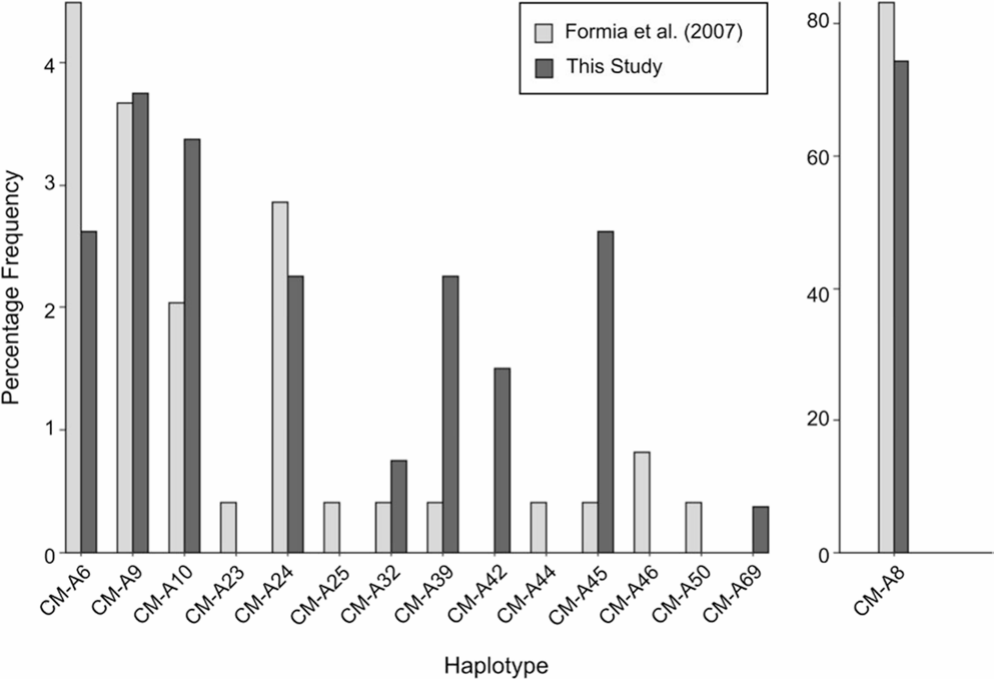
Genetic composition of green turtles (*Chelonia mydas*) from the Ascension Island rookery characterised by Formia et al. (2007; light grey) and this study (dark grey), using percentage frequencies for haplotypes based on a ~ 486 bp mitochondrial control region sequence (short D-loop). The dominant haplotype (CM-A8) is separated for ease of viewing

When comparing the ~ 486 bp shorter D-loop haplotypes, we found no significant difference between this study and the previous study on Ascension Island (Formia et al. 2007; *F*_*ST*_ = 0.00, *p* = 0.36, FDR corrected P value of 0.03). In general, the differences between the two studies were exhibited in rare haplotypes (Fig. 2). The haplotype CM-A8, dominant in the South Atlantic, was by far the most frequent in both studies (Formia et al. 2007: 83.3%; this study: 74.4%; Fig. 2). There were instances of haplotypes found only in our study (CM-A42, CM-A69), and haplotypes found only in the previous study (Formia et al. 2007; CM-A25, CM-A44, CM-A46, CM-A50; Fig. 2). Our study found increased frequencies of rare haplotypes CM-A45 and CM-A39, the percentage frequency having increased from 0.4 to 2.4% and 0.4 to 2.1%, respectively (Fig. 2).

Pairwise comparisons based on haplotype frequencies showed no significant differences among the three main Ascension Island nesting beaches (see Fig. 1 for names) when considering the extended D-loop (SWBxNEB: *F*_*ST=*_−0.010, *p* = 0.907; NEBxLB: *F*_*ST=*_0.000, *p* = 0.369; LBxSWB: *F*_*ST=*_*-*0.005, *p* = 0.583) or extended D-loop and mtSTR combined (SWBxNEB: *F*_*ST=*_0.015, *p* = 0.080; NEBxLB: *F*_*ST=*_0.002, *p* = 0.271; LBxSWB: *F*_*ST=*_0.025, *p* = 0.034) when using an FDR corrected P-value of *P* = 0.020.

**Table 3:** Sample size (n), haplotype number (hap), haplotype diversity (*h*, mean ± SD) of 21 green turtle (*Chelonia mydas*) Atlantic rookeries for haplotypes based on two genetic markers: (1) a ~ 738 bp mitochondrial control region sequence (extended D-loop); and (2) haplotypes based on a ~ 738 bp mitochondrial control region sequence combined with a ~ 200 bp region of short tandem repeats in the mtDNA control region (extended D-loop + mtSTR). The study population is shown in **bold**. Populations are ordered according to location, going anticlockwise around the Atlantic Ocean

Rookery		Extended D-loop			Extended D-loop + mtSTR		
		*n*	hap	h ± SD	*n*	hap	h ± SD
NUSA	Northern US Limit, USA	52	7	0.624 ± 0.044	-	-	-
CEFL	Central East Florida, USA	534	11	0.549 ± 0.016	534	29	0.724 ± 0.019
SEFL	South East Florida, USA	164	10	0.493 ± 0.044	164	25	0.859 ± 0.019
MKFL	Key West, Florida, USA	20	1	0.000 ± 0.000	20	2	0.100 ± 0.088
DTFL	Dry Tortugas, Florida, USA	67	6	0.591 ± 0.050	67	9	0.641 ± 0.048
EBCMX	Campeche and Yucatan, Mexico	173	5	0.264 ± 0.041	-	-	-
GUCB	Guanahacabibes Peninsula and San Felipe, Cuba	145	21	0.868 ± 0.018	-	-	-
IJCB	Isla de la Juventud, Cuba	9	3	0.556 ± 0.165	-	-	-
CLCB	Cayo Largo, Cuba	34	5	0.225 ± 0.094	-	-	-
CR	Tortuguero, Costa Rica	386	6	0.203 ± 0.026	386	23	0.524 ± 0.030
CI	Grand Cayman, Cayman Islands (Wild population), UK	57	12	0.575 ± 0.077	57	19	0.838 ± 0.037
BUC	Buck Island, USA	49	3	0.191 ± 0.072	-	-	-
AV	Aves Island, Venezuela	67	3	0.444 ± 0.061	-	-	-
SUR	Matapica and Galibi, Suriname	58	3	0.101 ± 0.054	-	-	-
FG	Cayenne, French Guiana	18	3	0.216 ± 0.124	13	6	0.782 ± 0.105
RA	Atol das Rocas, Brazil	37	7	0.466 ± 0.099	37	16	0.874 ± 0.043
FN	Fernando de Noronha, Brazil	16	2	0.233 ± 0.126	16	10	0.950 ± 0.031
TRI	Trindade Island, Brazil	99	7	0.640 ± 0.044	99	28	0.918 ± 0.016
**ASC**	**Ascension Island**,** UK**	**267**	**11**	**0.366 ± 0.038**	**199**	**33**	**0.802 ± 0.028**
STP	São Tomé and Príncipe	96	6	0.647 ± 0.042	74	19	0.830 ± 0.034
GB	Poilão, Guinea-Bissau	289	3	0.014 ± 0.010	288	6	0.083 ± 0.022

### Population structure

**Fig. 3:**
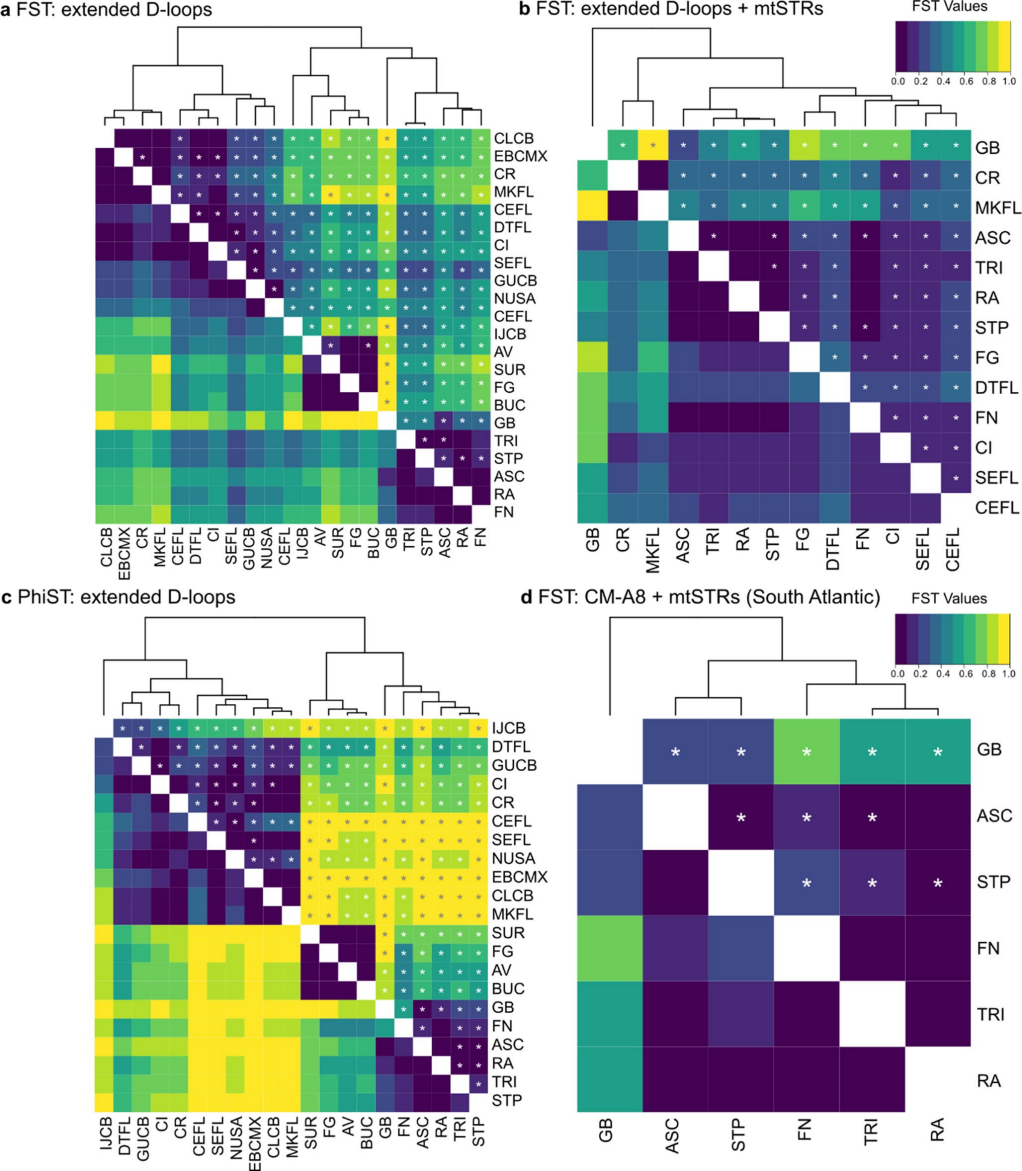
Heatmaps and dendrograms based on (a) *F*_*ST*_ pairwise comparisons for haplotypes based on the extended D-loop among 21 Atlantic green turtle nesting populations; (b) *F*_*ST*_ pairwise comparisons for haplotypes based on the extended D-loop and mtSTR among 13 Atlantic green turtle nesting populations; (c) *Ф*_*ST*_ pairwise comparisons for haplotypes based on the extended D-loop among 21 Atlantic green turtle nesting populations; (d) *F*_*ST*_ pairwise comparisons for CM-A8 + mtSTR among 6 South Atlantic rookeries. Asterisks are included in the above diagonal to indicate significant pairwise comparisons after FDR correction (for *P* ≤ 0.05, a) corrected *p* = 0.008; b) corrected *p* = 0.010; c) corrected *p* = 0.008; d) corrected *p* = 0.014; Narum 2006). See Table 1 for abbreviations

For the extended D-loop (*F*_*ST*_ and *Ф*_*ST*_), extended D-loop combined with mtSTR (*F*_*ST*_) and CM-A8 haplotypes combined with mtSTR (*F*_*ST*_), there was no significant genetic difference between Ascension Island (ASC) and Atol das Rocas (RA; Fig. 3, Table S6, Table S7, Table S8, Table S9). Ascension Island was also not significantly distinct from Fernando de Noronha (FN) for the D-loop when using *F*_*ST*_. Ascension Island was different to all other populations across the four comparisons.

**Fig. 4:**
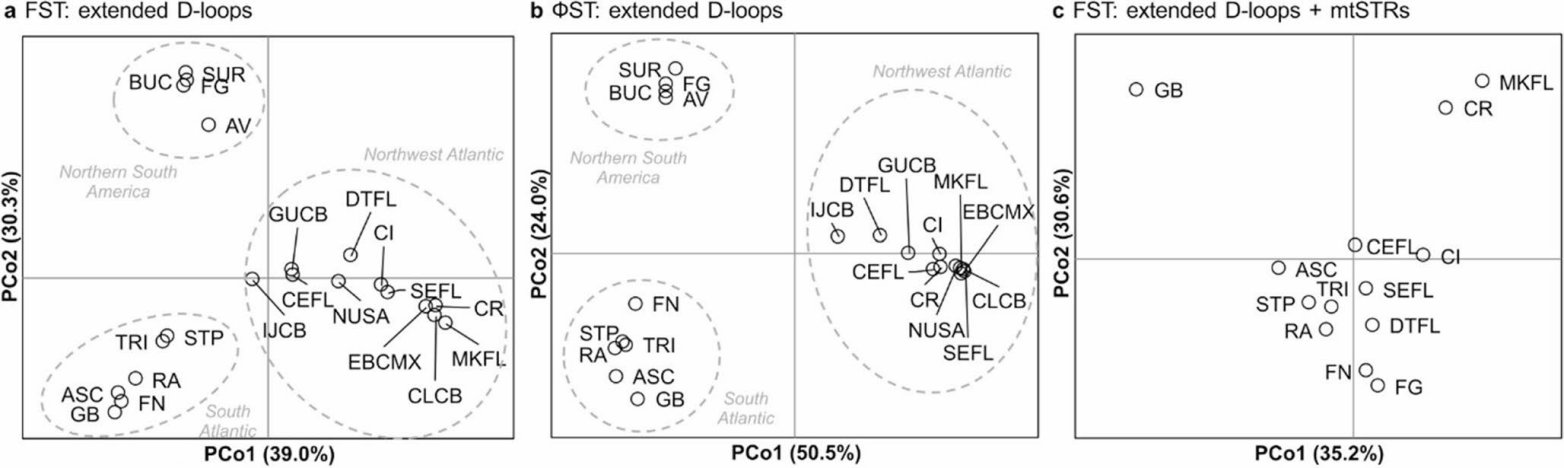
PCoAs based on (a) *F*_*ST*_ pairwise distances for haplotypes based on the extended D-loop among 21 Atlantic green turtle nesting populations; (b) *Ф*_*ST*_ pairwise distances for haplotypes based on the extended D-loop among 21 Atlantic green turtle nesting populations; (c) *F*_*ST*_ pairwise distances for haplotypes based on the extended D-loop + mtSTR among 13 Atlantic green turtle nesting populations. See Table 1 for abbreviations

The PCoA for both sets of markers separated the nesting populations into three main groups, each defined by a dominant haplotype: Northwest Atlantic (CM-A1/CM-A3), Northern South America (CM-A5), and South Atlantic and West Africa (CM-A8; Fig. 4). The principal coordinates explained 69.3% (*F*_*ST*_) and 74.5% (*Ф*_*ST*_) of genetic variability. An AMOVA using the a priori grouping derived from the PCoA based on *F*_*ST*_ and *Ф*_*ST*_ showed this grouping to be highly significant (*F*_*ST*_: *F*_*CT*_ = 0. 489, *P* = 0.000; *Ф*_*ST*_: *F*_*CT*_ = 0. 831, *P* = 0.000).

Principal coordinates of the PCoA based on the extended D-loop and mtSTR explained 65.8% of genetic variability. Visually, results do not adhere to traditional grouping; however, an AMOVA using this a priori grouping was significant (*F*_*CT*_ *=* 0.228, *P* = 0.017). Notably, it suggests that Guinea-Bissau is separated from the rest of the South Atlantic group.

### Mixed stock analyses

**Fig. 5:**
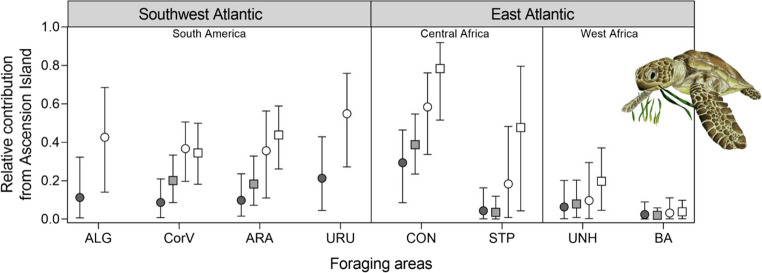
Relative contribution of Ascension Island green turtle (*Chelonia mydas*) rookery to eight Southwest Atlantic/East Atlantic juvenile foraging aggregations, estimated using rookery size as prior (white) and without priors (grey), using only extended D-loop haplotypes (circles) and with a combination of the extended D-loop and mtSTR (squares). See Table 1 for details on rookeries and foraging grounds incorporated and Table S10 for values

Mixed stock-centric results from many-to-many mixed stock analyses incorporating rookery size priors indicated that Ascension Island was the likely source population for a large proportion of juvenile green turtles at southwest Atlantic and Central African foraging sites (Fig. 5, Table S10).

The proportional contribution of Ascension to southwest Atlantic sites remained similar with and without incorporating the mtSTR (extended D-loop, 4 sites: range = 36–55%; extended D-loop + mtSTR, 2 sites: range = 34–44%) but increased considerably for the Central African sites (Fig. 5; extended D-loop: range = 18–58%; extended D-loop + mtSTR: range = 48–78%). Lower mean relative contributions were estimated for West Africa foraging aggregations, but the proportion increased when using the extended D-loop + mtSTR (extended D-loop: range = 3–10%; extended D-loop + mtSTR: range = 4–20%). Using priors substantially changed results. When the Ascension rookery size was not taken into account, contribution to all areas decreased (Fig. 5), yet the contribution of Ascension Island to southwest Atlantic and the Congo foraging aggregations remained important. Overall, a large proportion of the immature green turtles foraging in the South Atlantic seem to originate in Ascension Island (Fig. 5, Table S10).

## Discussion

To improve the understanding of green turtle connectivity in the Atlantic Ocean, we undertook a high-resolution genetic characterisation of the Ascension Island nesting population, using two mitochondrial genetic markers. We found novel haplotypes and uncovered important links between populations across the South Atlantic RMU.

### Genetic composition of Ascension Island

This study reinforces the importance of using higher-resolution markers in population genetics research. Using the mtSTR substantially increased the number of possible haplotype combinations by 230% compared to the shorter D-loop haplotype, theoretically improving the potential ability to detect genetic differentiation. No significant difference was shown among Ascension Island beaches, even when incorporating the highly polymorphic mtSTR, supporting the definition of Ascension Island as a single population both in our analyses and in its management actions. However, one comparison (South West Bay and Long Beach) was close to being significantly distinct when the mtSTR was incorporated. We found no significant difference in the genetic composition between our study and a previous genetic composition study of the island (Formia et al. 2007), despite being a decade apart. This suggests that our sample size was adequate and the population is stable.

Although overall differences between the two compositions were not significant, CM-A45 and CM-A39 increased in the present study compared to Formia et al. (2007) by a relatively large amount for rarer haplotypes, although this may be a sampling artefact. The present study recorded for the first time the extended D-loop haplotype containing the CM-A39 sequence previously found at Ascension Island. CM-A45 or CM-A39 have not been recorded in any other rookeries to our knowledge, but have been found in Brazilian, Argentinian and Uruguayan foraging grounds (e.g., Naro-Maciel et al. 2007; Proietti et al. 2012; Prosdocimi et al. 2024). This suggests that the haplotypes could be characteristic of Ascension Island and reinforces substantiated ties with Atlantic South American foraging grounds (Mortimer and Carr 1987; Luschi et al. 1998; Putman and Naro-Maciel 2013; Patrício et al. 2017a).

CM-A42, well-recorded in South American foraging grounds (e.g., Uruguay, Prosdocimi et al. 2024; Argentina, Prosdocimi et al. 2012; Brazil, Jordão et al. 2017), had previously only been identified in the rookery of Poilão, Guinea-Bissau (one individual; Patrício et al. 2017a). However, it had also been detected among green turtles from the Cayman Turtle Centre Ltd (Barbanti et al. 2019). Since Ascension Island was one of the main source populations of the Cayman founder stock, it was correctly hypothesised that CM-A42.1 was present in Ascension but remained to be discovered until this study (Barbanti et al. 2019). We also found for the first time at a rookery CM-A69 (CM-A69.1), which had previously been an ‘orphan’ haplotype found only at the São Francisco de Itabapoana foraging ground in Brazil (Jordão et al. 2017).

Ascension Island mtSTR haplotypes varied only in the first two repeat regions. For most of the haplotypes, the first region varied between 6 and 8 ‘AT’ repeats and the second region from 10 to 17, resembling the polymorphisms seen in characterisations of Brazilian rookeries (Shamblin et al. 2015b). However, we had two recordings of mtSTR sequences with 5 ‘AT’ repeats in the first region, an occurrence not found in these Brazilian rookeries, both in individuals with the CM-A45.1 haplotype, also not found in these rookeries. This finding suggests that despite the similarities between Ascension Island and the Brazilian rookeries shown by our population structure analyses, genetic distinctions may exist when considering rarer haplotypes. It also suggests that the combinations of haplotypes carry genetic structure, despite the fact that the mtSTR are more prone to homoplasy due to high mutation rates (Shamblin et al. 2015b).

### Atlantic green turtle population structure

The haplotype CM-A8 is dominant across South Atlantic populations (Naro-Maciel et al. 2007; Patrício et al. 2017a; Prosdocimi et al. 2024); however, using the mtSTR we were able to divide the CM-A8 haplotype into 18 mtSTR variations. The mtSTR haplotype 7-12-4-4 was dominant in almost all South Atlantic/West African rookeries studied. The combination of both the dominant haplotypes ‘CM-A8.1/7-12-4-4’ characterises the South Atlantic/West African populations.

PCoAs based on the extended D-loop indicated that three main groups of Atlantic rookeries could be clearly differentiated according to dominant haplotypes: Northwest Atlantic, Northern South America, and South and East Atlantic (as previously reported; Encalada et al. 1996; Patrício et al. 2017a). The high level of genetic similarity within the South and East Atlantic suggests frequent gene flow coupled with recent evolutionary history (Naro-Maciel et al. 2014). This may enhance basin-scale population genetic diversity (Slatkin 1987), particularly between Ascension Island and the Brazilian rookeries, given their genetic similarity across *F*_*ST*_, *Ф*_*ST*_ and D-loop/mtSTR analyses. Comparing structural analyses based on haplotype frequencies, the visually distinct Atlantic-wide structure (the three separated groups) is lost when the mtSTR was incorporated. The PCoA incorporating the mtSTR instead distinctly separates the nesting populations displaying the least genetic variability (Poilão, Guinea-Bissau; Key West, Florida [MKFL]; Tortuguero, Costa Rica). Within the less obviously separated group, the North Atlantic and South Atlantic populations are still separated by an axis, with the exception of Fernando de Noronha (FN). The D-loop marker, despite being less variable than the mtSTR, may incorporate variation more relevant to the population structure of green turtles in the study area. Its mutations could reflect historical separation, migration patterns, or breeding behaviours that have led to distinct genetic groupings (Encalada et al. 1996; Naro-Maciel et al. 2014). On the other hand, the high variability of the mtSTR, while potentially capturing more detailed genetic differences and separating recently genetically isolated populations (e.g., Tortuguero, Costa Rica and Poilão, Guinea-Bissau), does not seem to align as clearly with the population structure or historical separations. This high variability could introduce noise that obscures clear group distinctions in a PCoA. Hence, variation in the D-loop may be more relevant to oceanwide population structure across historically genetically varied groups on an Atlantic-wide scale, and the mtSTR may be a more suitable tool for further examining and refining population structure differences between historically similar populations which are dominated by the same D-loop haplotypes.

The lack of significant differentiation recorded between Ascension Island and Atol das Rocas across all four comparisons could suggest current gene flow facilitated by deviations in natal homing. Interestingly, Fernando de Noronha was significantly distinct from Ascension Island when comparing *Ф*_*ST*_ and when the mtSTR was included. Atol das Rocas is further from Ascension Island than Fernando de Noronha, although the Brazilian rookeries are only ~ 150 km from each other. Historically, Fernando de Noronha and Atol das Rocas were treated as a unit (Bjorndal et al. 2006). More recent, high-resolution mtSTR analysis suggested that they were discrete populations with respect to natal homing of females (Shamblin et al. 2015b), although conclusions were based on *Ф*_*ST*_ comparisons. *Ф*_*ST*_ accounts for sequence divergence and is now considered to be less appropriate than *F*_*ST*_ for the mtSTR due to the increased likelihood of different mutations leading to the same haplotype (Tikochinski et al. 2018; Shamblin et al. 2023a). Nevertheless, these results show that the mtSTR can reveal finer genetic differentiation between genetically similar populations.

### Green turtle connectivity in the Atlantic

**Fig. 6:**
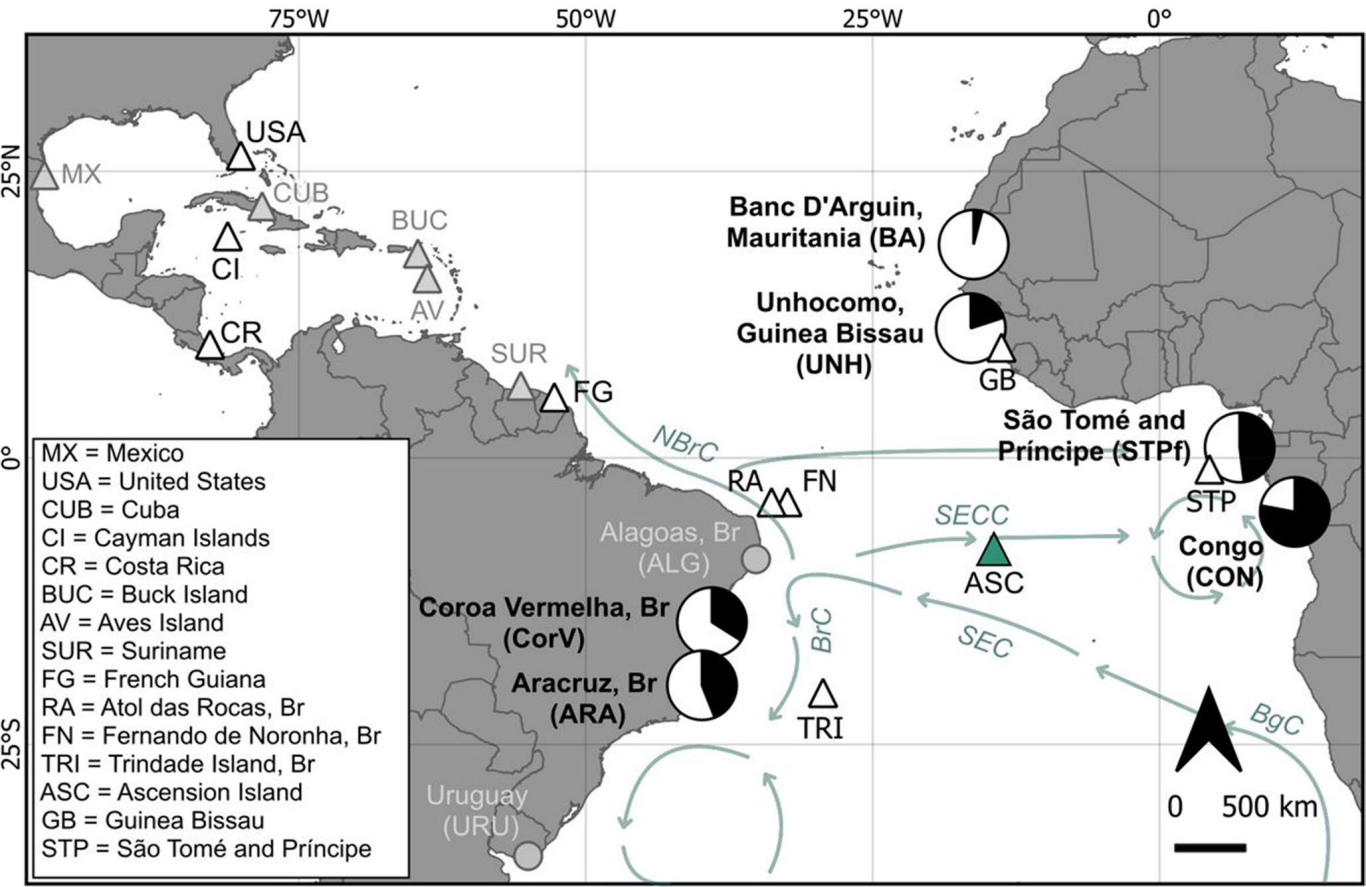
Map showing the contribution of the Ascension Island green turtle (*Chelonia mydas*) rookery (green triangle) to South Atlantic RMU mixed stock foraging grounds derived from many-to-many mixed stock analyses conducted on extended D-loop combined with mtSTR haplotype data from 10 Atlantic rookery groups and 6 mixed stock foraging aggregations. White triangles represent rookery groups, pie charts represent relative contributions from Ascension Island (black), estimated from an MSA with rookery size weighted priors. Greyed-out triangles and circles represent additional rookeries and foraging grounds included in the extended D-loop-only analysis. Arrows indicate the general direction of currents significant to the discussion, adapted from Muller-Karger et al. (2017). NBrC = North Brazil Current, SEC = South Equatorial Current, SECC = South Equatorial Countercurrent, BrC = Brazil Current, BgC = Benguela Current. See Table 1 for rookeries included within ‘MX’ and ‘USA’

Consistent with previous mixed stock analyses using shorter ~ 486 bp D-loop haplotypes (Bolker et al. 2007; Proietti et al. 2012; Jordão et al. 2017; Patrício et al. 2017a), our results based on the extended D-loop indicate that Ascension Island is a major source population for juvenile green turtle foraging aggregations in southern Brazil and further south in Uruguay (Fig. 6). We also show large contributions of Ascension Island to juvenile foraging areas in Central Africa (Congo and São Tomé and Príncipe), and, to a lesser extent, West Africa. When mtSTR haplotypes were included in the MSA analysis, contributions to the Southwest Atlantic foraging ground were similar, but those to Unhocomo (in Guinea-Bissau, West Africa), São Tomé and Príncipe and Congo showed a large increase. This suggests that the addition of the highly polymorphic mtSTR region may have increased the sensitivity of the analysis by reducing reliance on the CM-A8 haplotype. When population size was included as a prior in the MSAs, the estimated contributions from Ascension Island to these regions generally increased. Even when weighted priors were removed, the mean contributions from Ascension Island to the southwest Atlantic and Congo remained high, particularly when using the mtSTR marker. The notable exception was São Tomé and Príncipe, where the estimated mean proportion of turtles from Ascension dropped significantly when no priors were considered. This observation aligns with previous suggestions that the São Tomé and Príncipe population might be isolated, with foraging aggregations originating from the island’s own rookery, as discussed by Hancock et al. (2019).

Foraging aggregations from West Africa, particularly those in Mauritania, located further north, showed lower input from Ascension Island. Recent research estimated that green turtles foraging in Mauritania predominantly originate from Guinea-Bissau and Suriname or French Guiana (Patrício et al. 2024). In contrast, the foraging aggregations in Uruguay and Congo appear to rely heavily on recruits from Ascension Island. This strong connectivity with Uruguay was recently documented by Prosdocimi et al. (2024). Notably, even higher mean relative contributions were estimated to Congo. Ascension Island’s central location in the South Atlantic, at 7.947°S, places it directly within the South Equatorial Countercurrent (6°S-9°S) latitudes, which flows eastward. As a result, a significant proportion of post-hatchlings from Ascension Island may potentially drift with this current, while others are carried westward by the South Equatorial Current (Fig. 6; Brown 1990). The extent to which green turtle populations across the South Atlantic are connected by east-to-west (Patrício et al. 2017) and west-to-east (Monzón-Argüello et al. 2010) migrations has been an important and controversial question in recent research. Our results lend further support to this by demonstrating the connectivity of green turtles hatched at Ascension Island to both east and west. To date, no adult turtle has been satellite tracked migrating from Ascension Island to Central Africa, all have been tracked to Brazil, with recent tracking spanning the full nesting season (Luschi et al. 1998; Hays et al. 2002; S. Weber, personal communication November 4, 2024). We hypothesise that as juveniles, some Ascension Island green turtles may use developmental habitats on the Central African coast and then, as adults, migrate to foraging grounds in Brazil. The sampling of adults from Central African and Brazilian foraging grounds could help to confirm this theory by further characterising genetic links between these populations across life stages.

### Future directions

Our study reinforces the importance of using higher-resolution markers in marine turtle genetic analyses. The database of mtSTR sequences in Atlantic green turtles is less extensive than that of the D-loop. This additional marker may be key to identifying finer genetic differentiation, particularly within populations that have previously been treated as a single unit (Shamblin et al. 2015a, b). Additionally, targeted mitogenomic single nucleotide polymorphism (mtSNP) sequencing of the dominant CM-A8 haplotype may help to further increase resolution (Shamblin et al. 2023b), potentially revealing differences between Atol das Rocas and Ascension Island, and among Ascension Island’s beaches.

MSAs within the South Atlantic have previously been limited by the dominance of the CM-A8 haplotype. Our results incorporating the mtSTR suggest a greater link between Central and West Africa and the South Atlantic than when using D-loop haplotypes alone. However, for a more robust conclusion, more populations need to be characterised for mtSTR sequences, and some populations require larger sample sizes, namely French Guiana (nesting), and São Tomé and Príncipe (nesting and foraging). Bioko, in particular, is a key Central African rookery, not yet characterised via the extended D-loop or mtSTR, that likely has important genetic relationships with the populations discussed in this study (Formia et al. 2006). Confidence intervals in our MSA were large, as has been noted in several MSA studies on Atlantic green turtles (e.g., Proietti et al. 2012; Stahelin et al. 2022), and so results need to be treated with caution as the exactness of estimates is uncertain. These large intervals may be the result of incomplete representation of source rookeries or high regional gene flow in the Atlantic (Naro-Maciel et al. 2014; Patrício et al. 2024). Increased sample sizes among and within populations have the potential to improve the reliability of MSA estimates. There is a particular need for larger sample sizes when using the mtSTR to produce robust baseline frequencies due to their highly polymorphic nature (Shamblin et al. 2015b). Additionally, incorporating the distance between rookeries and foraging grounds, in combination with nester abundance, has been shown to improve the reliability of MSA estimates (Stahelin et al. 2022; Dolfo et al. 2023), and should thus be considered in future assessments.

Mitochondrial heteroplasmy also raises some important questions in our study. Some level of heteroplasmy was present in all our mtSTR sequences. Whilst most haplotypes could be determined based on the dominant variant using relative peak heights (Tikochinski et al. 2012; Shamblin et al. 2020), several had to be excluded due to the inability to determine the dominant mtDNA haplotype present. High-throughput sequencing or genotyping could improve haplotype determination in heteroplasmic individuals and prevent the loss of samples from the dataset, as well as identify further heteroplasmic haplotypes beyond the two afforded by Sanger sequencing (Tikochinski et al. 2020). Despite occurrences of heteroplasmy, mtSTRs have been shown to be robust to identify population structure and continue to be used in Mediterranean studies (Tikochinski et al. 2018; Karaman et al. 2022; Ohana et al. 2025). High amounts of heteroplasmy have also been reported in the Atlantic, where dominant haplotypes were assigned in ‘virtually all cases’ (Shamblin et al. 2020). Recently, it has been proposed that heteroplasmy in sea turtles may provide an evolutionary advantage by improving population diversity and acting as a buffer to population bottlenecks (Tikochinski et al. 2020). This suggests that comparing levels of mitochondrial heteroplasmy, looking at differences between regions and even life stages, could be relevant when mapping the trajectory of Atlantic green turtle populations in response to current threats.

### Conservation implications

Whilst Ascension Island’s nesting grounds and the surrounding waters are legally protected and the population is in recovery from a dramatic reduction caused by mass harvesting for consumption (Weber et al. 2014), the dependence of breeding green turtles on other areas of the Atlantic during their lifecycle and migrations exposes them to a range of threats. For instance, fishery bycatch within artisanal gillnets is a significant threat to green turtles along the Brazilian coast (López-Barrera et al. 2012) and the west coast of Africa (Catry et al. 2009; Moore et al. 2010). Here, we show important links between Ascension Island and juvenile foraging areas along the South American, West African and Central African coasts, highlighting the need for international collaborations between nations that have a shared responsibility for this turtle population. Such collaborations could lead to the establishment of coordinated regional monitoring programmes, the exchange of crucial demographic information (such as genetic makeup), and the dissemination of reports on prevalent threats (including bycatch rates and poaching incidents). They could also offer an avenue for facilitating the transfer of knowledge and skills, as well as enabling the analysis of data collected by less financially resourced programs through the support of better-funded partners. Moreover, regional cooperation can significantly amplify efforts and exert greater pressure on stakeholders to implement effective conservation measures at sites that are interconnected through this turtle population and face significant threats.

## Supplementary Information

Below is the link to the electronic supplementary material.

ESM 1 (XLSX 103 KB)

## Data Availability

Data supporting the findings of this study can be found within the article and Supplementary material. Novel haplotype sequence data can be found in Genbank under accession numbers PQ604655 and PP429908.
